# Editorial: Proteomics for Studying Foodborne Microorganisms and Their Impact on Food Quality and Human Health

**DOI:** 10.3389/fnut.2019.00104

**Published:** 2019-07-10

**Authors:** Rosa Anna Siciliano, Sergio Uzzau, Maria Fiorella Mazzeo

**Affiliations:** ^1^Institute of Food Sciences, National Research Council (CNR-ISA), Avellino, Italy; ^2^Department of Biomedical Sciences, University of Sassari, Sassari, Italy

**Keywords:** proteomics, microorganisms, food quality, food, human health

Foodborne microorganisms may play a pivotal role in mediating the tight relation between food and human health from a dual perspective. In fact, notwithstanding the introduction of strict regulations and new technologies to ensure food quality and safety, foodborne pathogens continue to cause infections and diseases, representing a serious public health concern, while spoilage bacteria can severely affect food quality thus leading to major industry and commercial losses.

On the other hand, probiotics positively affect human health, promoting digestion and uptake of dietary nutrients, strengthening intestinal barrier function, modulating immune response, and enhancing antagonism toward pathogens.

The present Research Topic of Frontiers in Nutrition was aimed to bring together research findings that further assess the strength of proteomics in exploring different aspects of the foodborne microorganisms' world.

Proteomics represents a key discipline to investigate physiology of foodborne bacteria since it enables the most accurate identification of complex networks of proteins underlying beneficial or harmful effects and cellular adaptation to different growth conditions, such as low temperatures. In this regard, refrigeration is still a widely used method for food storage and bacterial acclimation can favor food contamination and spoilage.

In their exemplary study, Santos et al. characterized the surface proteins of *Listeria monocytogenes*, grown in biofilm, to explore cold stress adaptation mechanisms boosted by this microorganism. In fact, capability to survive and proliferate at low temperature represents an essential feature of this foodborne pathogen enabling contamination of refrigerated and ready-to-eat foods.

Similarly, the cold tolerance features of *Vibrio parahaemolyticus*, a foodborne pathogen commonly isolated in seafood, have been elucidated. The integration of transcriptomic and proteomic data, collected from frozen squid and clinical isolates revealed the role of pyruvate metabolism in the processes of cold adaptation (Xie et al.).

This interesting microorganism is also able to survive in a viable but not culturable (VBNC) state when exposed to adverse conditions, such as refrigeration, and it can regain culturability (resuscitation state) under appropriate conditions. The molecular processes involved in this resuscitation process have been investigated by Zhong et al.

The stress response mechanisms of bacteria are tightly regulated at transcriptional level and RpoS is known as an alternative sigma factor controlling stress resistance and virulence in many pathogens. Liu et al. defined the regulatory network of RpoS and assessed its role in biofilm formation and spoilage potential of *Pseudomonas fluorescens*, a bacterium often responsible for the deterioration of proteinaceous foods.

The development of innovative methods for the detection of pathogens and spoilage bacteria is still an indispensable research issue that can successfully take advantage of proteomic methodologies. As reported in this Research Topic, immune-proteomics was applied by Shi et al. to identify cell surface associated proteins in *Alicyclobacillus acidoterrestris*, a major putrefying bacterium mainly contaminating juice, and to select antigens potentially useful for the development of an immunological method for the detection of this contaminant.

Interestingly, different strains of the same bacterial species can exhibit either probiotic or pathogenic features. As matter of fact, *Enterococcus faecalis* is a controversial microorganism since, on one side, different strains can be used as food starters and probiotics, while, on the other, *E. faecalis* clinical isolates are now considered emergent nosocomial pathogens, exhibiting resistance to antibiotics and putative virulence factors. The high-throughput proteomic study performed on three different strains of this microorganism by Cirrincione et al. provided additional evidence to assess their food safety and highlight potential probiotic or pathogenic traits.

To gain a thorough view of the molecular mechanisms associated to probiotic or pathogenic features of bacteria, it is of the outmost importance the analysis of the surface exposed proteins that represent the front-line of interaction of the bacterial cells with their environmental niches. In the last decade, *ad hoc* designed proteomic strategies have been developed to analyze this complex class of molecules, taking advantages of the recent technological improvements in mass spectrometry and bioinformatics and thus providing direct evidence of protein cell localization and cell wall topology. These methods and the most updated and relevant results in the probiotics field have been reviewed by Siciliano et al.

In this frame, the analysis of the surfaceome of the well-known probiotic strain *Lactobacillus rhamnosus* GG highlighted that different growth conditions (glucose or fructose as carbon source of cells grown in planktonic or biofilm state) qualitatively and quantitatively modified the pattern of surface exposed proteins thus affecting probiotic (adherence and immunomodulatory) and industrially relevant (proteolytic activity) features (Savijoki et al.).

On the other hand, surface proteins are directly involved in bacteria pathogenicity and virulence and, for instance, in *Salmonella enterica* subspecies *enterica* (SEE), these features can vary considerably across serovars (and even strains). In this context, Fagerquist and Zaragoza confirmed the presence of flagella proteins in the surfaceome of three different SEE serovars and that of secreted invasion/effector proteins (Sip) in the Newport and Thompson strains.

In conclusion, the articles gathered in this Research Topic offer an overview of the most up to date proteomic methodologies and further highlight the relevance of this discipline in exploring different aspects of the foodborne microbial lifestyle, enabling the definition of microbial biomarkers of food quality and safety and, in turn, the design of new food preservative strategies.

We hope that this Research Topic will be interesting for the readers of the journal and we are grateful to all authors, reviewers and editorial staff who contributed to the realization of this initiative.

## Author Contributions

All authors listed have made a substantial, direct and intellectual contribution to the work, and approved it for publication.

### Conflict of Interest Statement

The authors declare that the research was conducted in the absence of any commercial or financial relationships that could be construed as a potential conflict of interest.

